# GBP3-STING interaction in glioblastoma coordinates autophagy, anti-oxidative, and DNA repair programs in response to temozolomide

**DOI:** 10.18632/oncotarget.28370

**Published:** 2023-05-19

**Authors:** Jun Ma, Ziyu Wang, Clark C. Chen, Ming Li

**Keywords:** GBP3, MGMT, glioblastoma, temozolomide resistance, STING

With the introduction of the methylating agent, Temozolomide (TMZ), as the standard adjuvant chemotherapeutic drug for glioblastoma [1], the survival rate of this WHO Grade IV tumor which constitutes 17.7% [2] of overall primary central nervous system tumors has achieved a clinically significant prolongation of 2.5 months overall and an increase of 16.3% 2-year survival rate [3]. However, one intractable challenge is the diverse reactions to temozolomide treatment. The acquired resistance to the standard adjutant radiochemotherapy including temozolomide has favored the recurrence of some glioblastoma cases and has kept the milestone from moving forward for more than 15 years.

Guanylate-binding proteins (GBPs) are a group of dynamin-related large (~65 kDa) GTPases expressed in response to interferon and mediate intracellular immunity. Consisting of 7 members in human, little is known about the function of GBPs beyond their role in innate cellular immunity. After recent years of dedication on GBP family, its role in glioblastoma’s development and recurrence has drawn great attention, showing that the GBP family members such as GBP1, GBP2, GBP3, and GBP5 are highly elevated and play pro-tumor roles through multiple mechanisms in glioblastoma [4–8]. More recently Li’s lab did informatic analysis of clinically annotated glioblastoma datasets, laboratory studies of protein-protein interaction, and functional characterization after depletion or exogenous expression. Although other GBP family members did not show prominent relationship with treatment resistance at the present stage, GBP3 showed significant up-regulation in response to temozolomide [9]. Furthermore, it’s revealed that high levels of GBP3 expression in glioblastoma was associated with worsened survival after temozolomide treatment. Consistent with this observation, exogenous expression of GBP3 induced temozolomide resistance in independent patient-derived glioblastoma neurosphere lines, while GBP3 silencing conferred temozolomide sensitivity, both *in vitro* and *in vivo*. This sensitivity was associated with the accumulation of cytoplasmic DNA fragments, suggesting the involvement of Stimulator of interferon genes (STING). Cytoplasmic DNA bound STING usually activates key transcriptional programs. By profiling transcription programs requiring both GBP3 and STING expression, the autophagy program mediated by p62, the defense program against oxidative insults mediated by nuclear factor erythroid 2 like 2 (NFE2L2, NRF2), and the DNA repair program mediated by O6-methylguanine-DNA-methyltransferase (MGMT) were identified [9]. These programs were previously shown to play central roles in cellular resistance to DNA alkylating agents [10]. It turned out that the N-terminal of GBP3 physically interacted with STING to prevent proteasome-mediated degradation of STING. Through the GBP3/STING-p62-NRF2-MGMT cascade, the coordination of autophagy, anti-oxidative, and DNA repair programs, GBP3 could mediate cellular response to DNA damage induced by TMZ. It decreases DNA damage, enhances DNA damage repair, thus regulates TMZ resistance in glioblastoma cells.

Besides the effort on revealing the mechanisms that are responsible for glioblastoma initiation and growth, another main effort has been consistently put on counteracting the resistance to therapies to improve the overall prognosis for all glioblastoma patients. Facing radiochemotherapy induced toxic stress, glioblastoma cells undergo DNA damage repair to attenuate cytotoxicity, autophagy to save energy, stemness to grant regrowth, senescence to trigger transformation favorable inflammatory microenvironment, etc. [11]. These mutations help glioblastoma cells evade radiation and TMZ induced apoptosis and develop treatment resistance. The paper revealed a critical role of GBP3 involving multiple TMZ resistance mechanisms. This will be adding potential novel prognostic markers and, more importantly, new therapy targets for glioblastoma. The future is unlimited.
